# Complement activation and cellular inflammation in Fabry disease patients despite enzyme replacement therapy

**DOI:** 10.3389/fimmu.2024.1307558

**Published:** 2024-01-18

**Authors:** Björn Laffer, Malte Lenders, Elvira Ehlers-Jeske, Karin Heidenreich, Eva Brand, Jörg Köhl

**Affiliations:** ^1^ Institute for Systemic Inflammation Research, University of Lübeck, Lübeck, Germany; ^2^ Department of Internal Medicine D, University Hospital Münster, Münster, Germany; ^3^ Eleva GmbH, Freiburg, Germany; ^4^ Division of Immunobiology, Cincinnati Children’s Hospital Medical Center, Cincinnati, OH, United States

**Keywords:** Fabry disease, complement, C5a, C3a, enzyme replacement therapy

## Abstract

Defective α-galactosidase A (AGAL/GLA) due to missense or nonsense mutations in the *GLA* gene results in accumulation of the glycosphingolipids globotriaosylceramide (Gb3) and its deacylated derivate globotriaosylsphingosine (lyso-Gb3) in cells and body fluids. The aberrant glycosphingolipid metabolism leads to a progressive lysosomal storage disorder, i. e. Fabry disease (FD), characterized by chronic inflammation leading to multiorgan damage. Enzyme replacement therapy (ERT) with agalsidase-alfa or -beta is one of the main treatment options facilitating cellular Gb3 clearance. Proteome studies have shown changes in complement proteins during ERT. However, the direct activation of the complement system during FD has not been explored. Here, we demonstrate strong activation of the complement system in 17 classical male FD patients with either missense or nonsense mutations before and after ERT as evidenced by high C3a and C5a serum levels. In contrast to the strong reduction of lyso-Gb3 under ERT, C3a and C5a markedly increased in FD patients with nonsense mutations, most of whom developed anti-drug antibodies (ADA), whereas FD patients with missense mutations, which were ADA-negative, showed heterogenous C3a and C5a serum levels under treatment. In addition to the complement activation, we found increased IL-6, IL-10 and TGF-ß1 serum levels in FD patients. This increase was most prominent in patients with missense mutations under ERT, most of whom developed mild nephropathy with decreased estimated glomerular filtration rate. Together, our findings demonstrate strong complement activation in FD independent of ERT therapy, especially in males with nonsense mutations and the development of ADAs. In addition, our data suggest kidney cell-associated production of cytokines, which have a strong potential to drive renal damage. Thus, chronic inflammation as a driver of organ damage in FD seems to proceed despite ERT and may prove useful as a target to cope with progressive organ damage.

## 1 Introduction

Fabry disease (FD; Online Mendelian Inheritance in Man [OMIM] no. 301500) is an X-linked inherited disorder caused by a lack or reduced activity of the lysosomal enzyme α-galactosidase A (AGAL; EC 3.2.1.22) activity. AGAL deficiency results in a lysosomal accumulation of the enzyme substrate Gb3 in most cell types of affected patients, including cardiac, renal, neuronal and vascular cells. The progressive accumulation leads to a multisystemic disorder with early cerebrovascular events, heart failure, cardiac arrhythmia, and end-stage renal disease with a mean reduction of lifespan by 10 to 15 years (1).

FD is mainly classified into classic or late-onset subtypes. Very low AGAL activity levels (<1% of normal) or the complete loss-of-function are associated with classic FD, whereas residual enzyme frequently leads to slow disease progression and a milder late-onset phenotype (2, 3). Among the >900 FD mutations (http://www.hgmd.cf.ac.uk/ac/gene.php?gene=GLA; http://fabry-database.org) (4, 5), nonsense mutations account for ~11% of all described disease-causing *GLA* gene mutations (6). They are defined by premature termination codons leading to a complete loss-of-function of AGAL and a more severe phenotype. Missense mutations alter the amino acid composition of the enzyme often resulting in misfolded and unstable proteins with reduced intracellular AGAL activity (7, 8).

Standard treatment for FD patients includes intravenous ERT with agalsidase-alfa, agalsidase-beta or oral chaperone therapy (9). FD-specific treatment effectively reduces intracellular Gb3 and plasma lyso-Gb3 levels, associated with an improved clinical outcome (10–14). However, especially patients with more advanced disease progression seem to benefit less from the current therapeutic options. The available data suggest that AGAL deficiency triggers cellular inflammatory processes (15, 16) leading to endoplasmatic reticulum (ER), mitochondrial and oxidative stress, and cellular dysregulation at the level of the lysosomal pathway, endocytosis and autophagy (16, 17). At this point, it remains unclear to what extent FD-specific ERT and chaperone approaches affect and regulate humoral inflammatory processes, in particular activation of the complement system. Further, ERT can lead to formation of neutralizing ADAs in the early course of disease that may attenuate therapy efficacy and mediate disease progression in affected male patients (18–21).

Recently, we found a strong IgG autoantibody-induced complement activation and C5a generation in Gaucher disease (GD), an autosomal recessive inherited disorder caused by dysfunction of the lysosomal enzyme β-glucosidase leading to extensive accumulation of glucosylceramide (GC) (22). Activation of C5a receptor 1 stimulated the inflammatory response through upregulation of UDP-glucose ceramide glucosyltransferase (GCS), which is also critical for the formation of Gb3.

Here, we assessed humoral und cellular inflammation in male patients with classic FD before and under ERT treatment. For this purpose, we determined serum concentrations of the anaphylatoxins C3a and C5a reflecting complement activation as well as serum concentrations of several pro- and anti-inflammatory cytokines and chemokines mirroring cellular activation.

## 2 Material and methods

### 2.1 Patients and blood samples

Patients were treated with agalsidase-alfa (0.2 mg/kg body weight, every other week) or agalsidase-beta (1.0 mg/kg bodyweight every other week; both intravenously). Written informed consent was obtained from the participants for analysis and publication. The estimated glomerular filtration rate (eGFR) was quantified using the Chronic Kidney Disease-Epidemiology Collaboration (CKD-EPI)-based equation on the basis of serum creatinine (23). Albuminuria was defined as an albumin-creatinine-ratio (ACR) >30 mg albumin per gram of creatinine from spot urine. Hypertension was defined as a mean blood pressure of ≥135/85 mmHg during the day and/or ≥120/70 mmHg at night, as determined by 24-hour blood pressure measurement (24). The presence of left ventricular hypertrophy (LVH) was defined as an interventricular septum thickness in diastole >11.5 mm (25). Disease severity was assessed as total MSSI (26) and DS3 (25) scores. The presence of neutralizing anti-drug antibodies (ADAs) was assessed according to our standard protocols (21, 27). Plasma lyso-Gb3 (reference value ≤1.8 ng/ml) and *GLA* mutations were assessed by Centogene (Rostock, Germany).

### 2.2 Blood sampling

Venous blood was collected using 7.5-ml S-monovettes (serum gel-stabilized for serum and EDTA-stabilized for plasma; Sarstedt). Blood was centrifuged for 10 min at 2,000 x g and serum/plasma samples were stored at -80°C until analyzed. Serum samples for determination of C3a, C5a, cytokines and chemokines were collected from the Department of Internal Medicine D, and Interdisciplinary Fabry Center (IFAZ), University Hospital Münster (FD patients) and University Medical Center Schleswig-Holstein, Lübeck (healthy volunteers).

### 2.3 Complement assays

For quantification of complement cleavage products C3a/C3a-desArg (C3a) as well as C5a/C5a-desArg (C5a) in serum, commercial C3a (HK354, Hycult Biotech, Uden, Netherlands) and C5a (HK349, Hycult Biotech, Uden, Netherlands) human enzyme-linked immunosorbent assay (ELISA) kits were used according to the manufacturer’s instructions. Sera from FD patients were diluted 1:30,000 to 1:100,000 (C3a) or 1:10 to 1:30 (C5a). Sera from healthy controls were diluted 1:3,000 to 1:10,000 (C3a) or 1:5 to 1:10 (C5a). Samples were run in duplicates.

### 2.4 Immunoassays

For serum cytokine quantification, including IL-1β, TNF-α, IL-17A, IL-6, IL-10, IFN-γ and TGF-β1 (Free Active), we used the bead-based multiplex LEGENDplexTM assay (LEGENDplexTM Human Essential Immune Response Mix and Match Subpanel, Biolegend, CA, USA) according to the manufacturer’s instructions. For serum chemokine quantification including CC chemokine ligands (CCL) CCL2 (MCP-1), CCL3 (MIP-1α), CCL17 (TARC) and C-X-C motif chemokine ligands (CXCL) CXCL1 (GROα), CXCL8 (IL-8), CXCL10 (IP-10), we used the bead-based multiplex LEGENDplexTM assay (LEGENDplexTM Human Proinflammatory Chemokine Panel 1 Mix and Match Subpanel, Biolegend, CA, USA) according to the manufacturer’s instructions. Sera from healthy controls and FD patients were diluted 1:2. Reactions were performed in duplicates. Analysis was performed with the Cytek Aurora flow cytometer (Cytek Biosciences, CA, USA) supported by CAnaCore (Cell Analysis Core Facility, Lübeck, Germany). Data were analyzed via LEGENDplex™ Data Analysis Software (BioLegend in agreement with Qognit, CA, USA) and specified as pg/ml.

### 2.5 Statistical analysis

For statistical analysis, GraphPad PRISM 9 software was used. The data obtained were analyzed for normal distribution by the Kolmogorov-Smirnov test. Differences between two groups (controls vs treatment-naïve, FD patients with missense vs. nonsense mutations or ADA-negative vs. ADA-positive FD patients) were determined by either unpaired Student t-test (parametric) or Mann-Whitney rank sum test (nonparametric). Differences in serum levels of anaphylatoxins, cytokines or chemokines before (V0) and under (V1) ERT were evaluated by paired Student t-test. For comparisons of multiple groups, a one-way ANOVA with Tukey’s posthoc test (parametric) or Kruskal-Wallis-test with Dunn’s posthoc test (nonparametric) was performed. Correlations between two parameters were assessed by linear regression analysis. Differences were considered as significant with a p-value <0.05.

## 3 Results

### 3.1 Clinical characteristics of the FD patient cohorts

The study population consisted of 24 male treatment-naïve classical FD patients and 18 healthy male control subjects. Seventeen of the treatment-naïve FD patients were followed-up 12 to 104 months after the initiation of ERT. The mean age of the control group (53 ± 19 years) was comparable to the 24 treatment-naïve FD patients (43 ± 19 years).

The baseline characteristics of the 24 ERT-naïve patients are shown in **Table 1**. Fifteen patients carried missense and nine patients nonsense mutations (**Table 2**). Most baseline characteristics were comparable between the patients with missense and nonsense mutations. Kidney function was not compromised in the two patient groups. Sixty percent of the FD patients with missense mutations but none of the FD patients with nonsense mutations suffered from hypertension (p<0.0068) and were treated with RAAS-blockers. The presence of left ventricular hypertrophy in patients with nonsense and missense mutations was comparable (64.3% and 62.5%, respectively). Both patient groups showed similar disease severity as evidenced by comparable DS3 and MSSI scores. In 21 of the 24 treatment-naïve FD patients, plasma lyso-Gb3 levels were above the reference values. Plasma lyso-Gb3 levels in treatment-naïve FD patients with nonsense mutations were higher (128.9 ± 30.6 ng/ml) as compared to those with missense mutations (38.0 ± 40.4 ng/ml; p=0.0001).

**Table 1:** Baseline characteristics of treatment-naïve FD patients.

	total [n=24]	missense mutations [n=15]	nonsense mutations [n=9]	p-value
**age, years**	42.6 ± 19.0	47.7 ± 19.8	34.0 ± 14.8	0.0865
**lyso-Gb_3_, ng/ml**	72.1 ± 57.8	38.0 ± 40.4	128.9 ± 30.6	**0.0001**
**lyso-Gb_3_ above the reference, n**	21 (87.5)	12 (80.0)	9 (100.0)	0.2663
**serum creatinine, mg/dl**	1.13 ± 0.83	1.25 ± 1.03	0.91 ± 0.27	0.3436
**eGFR, ml/min/1.73 m²**	96.1 ± 37.3	88.7 ± 40.5	108.3 ± 29.3	0.2193
**ACR, mg/g**	56 [0-6767]	57 [0-6767]	56 [0-1915]	0.9391
**albuminuria, n**	15 (65.2)	8 (57.1)	7 (77.8)	0.3998
**dialysis, n**	0 (0.0)	0 (0.0)	0 (0.0)	0.9999
**septum thickness, mm**	13.4 ± 4.3	13.7 ± 4.9	12.8 ± 3.1	0.6590
**LVH, n**	14 (63.6)	9 (64.3)	5 (62.5)	0.9999
**atrial fibrillation, n**	4 (16.7)	3 (20.0)	1 (11.1)	0.9999
**myocardial infarction, n**	1 (4.2)	0 (0.0)	1 (11.1)	0.3750
**hypertension, n**	9 (37.5)	9 (60.0)	0 (0.0)	**0.0068**
**stroke/TIA, n**	3 (12.5)	3 (20.0)	0 (0.0)	0.2663
**RAAS blockers, n**	11 (45.8)	10 (66.7)	1 (11.1)	**0.0131**
**diuretics, n**	2 (8.3)	2 (13.3)	0 (0.0)	0.5109
**anticoagulants, n**	4 (16.7)	3 (20.0)	1 (11.1)	0.9999
**platelet aggregation inhibitors, n**	4 (16.7)	2 (13.3)	2 (22.2)	0.6146
**DS3 total, score**	16.4 ± 9.7	18.5 ± 8.8	13.3 ± 10.6	0.2315
**MSSI total, score**	18.0 ± 10.8	19.0 ± 11.1	16.4 ± 10.7	0.5964

Values are given as mean ± SD or median [range], if non-normally distributed. The frequency (%) is given in parenthesis. Platelet aggregation inhibitors include acetylsalicylic acid or clopidrogel. Anticoagulants include treatment with vitamin K antagonists (VKAs) or direct oral anticoagulants (DOACs/NOACs). Statistically significant differences between groups are shown in bold. ACR, albumin-creatinine ratio; ADA, anti-drug antibodies; DS3, Disease Severity Score System; eGFR, estimated glomerular filtration rate (CKD-EPI-based); ERT, enzyme replacement therapy; LVH, left ventricular hypertrophy defined as septum thickness >11.5 mm; lyso-Gb_3_, globotriaosylsphingosine (reference value ≤1.8 ng/ml); MSSI, Mainz Severity Score Index; RAAS, renin-angiotensin-aldosterone-system.

**Table 2:** Overview of the identified α-galactosidase A mutations in the Fabry disease patients.

missense mutations [n=15]	nonsense mutations [n=9]
p.G35E, p.L45P, p.I91T, p.R112C, p.A143T (2x), p.A160P, p.C202Y, p.N215S (3x), p.I242V, p.G274D, p.P301Q, p.R342Q	*single nucleotide stop codon mutations* p.Y151X, p.Y216X, p.R227X, p.W349X *insertions/deletions* c.702_del8bp, c.714_715ins, c.723dupT, c.744_745del, c.762ins282bp

Clinical parameters (baseline and follow-up) of the 17 followed-up ERT-treated patients are shown in **Tables 3**, **4**. FD patients carrying missense (n=9) or nonsense (n=8) mutations showed no major differences regarding most clinical parameters except plasma lyso-Gb3 levels, which were still significantly higher in FD patients with nonsense mutations (34.6 ng/ml [range: 18.6-101.0] as compared with those with missense mutations (19.0 ng/ml [range: 0.6-41.0]). In fact, in 16 of the 17 patients lyso-Gb3 levels were still above the reference value after ERT initiation. Further, patients with missense mutations showed a reduced eGFR (68.4 ± 43.9 ml/min/1.73 m²) demonstrating mild renal impairment (chronic kidney disease [CKD] stage 2). This impairment of kidney function is partly due to the older age of patients with missense mutations as compared to patients with nonsense mutations (28) (**Table 3**). Finally, all FD patients with nonsense mutations developed neutralizing antibodies against recombinant α-galactosidase A, whereas only three of the nine FD patients with missense mutations developed neutralizing ADAs (33.3%).

**Table 3:** Baseline characteristics of the 17 ERT-treated patients.

	total [n=17]	missense mutations [n=9]	nonsense mutations [n=8]	p-value
**age, years**	40.9 ± 18.5	48.4 ± 18.8	32.5 ± 15.0	0.0752
**lyso-Gb_3_, ng/ml**	85.3 ± 50.8	51.4 ± 41.8	123.4 ± 27.6	**0.0009**
**lyso-Gb_3_ above the reference, n**	17 (100.0)	8 (88.9)	8 (100.0)	0.9999
**serum creatinine, mg/dl**	1.22 ± 0.97	1.51 ± 1.28	0.89 ± 0.28	0.4965
**eGFR, ml/min/1.73 m²**	94.1 ± 40.0	78.6 ± 43.1	111.6 ± 29.4	0.0745
**ACR, mg/g**	114 [0 to 6,767]	679 [0 to 6,767]	55 [0 to 1915]	0.1315
**albuminuria, n**	14 (82.4)	8 (88.9)	6 (75.0)	0.5765
**dialysis, n**	0 (0.0)	0 (0.0)	0 (0.0)	0.9999
**septum thickness, mm**	13.7 ± 4.0	14.8 ± 4.6	12.5 ± 3.2	0.3000
**LVH, n**	10 (58.8)	6 (66.7)	4 (50.0)	0.6372
**atrial fibrillation, n**	3 (17.6)	2 (22.2)	1 (12.5)	0.9999
**myocardial infarction, n**	1 (5.9)	0 (0.0)	1 (12.5)	0.4706
**hypertension, n**	6 (35.3)	6 (66.7)	0 (0.0)	**0.0090**
**stroke/TIA, n**	2 (11.8)	2 (22.2)	0 (0.0)	0.4706
**RAAS blockers, n**	8 (47.1)	7 (77.8)	1 (12.5)	**0.0152**
**diuretics, n**	2 (11.8)	2 (22.2)	0 (0.0)	0.4706
**anticoagulants, n**	4 (23.5)	3 (33.3)	1 (12.5)	0.5765
**platelet aggregation inhibitors, n**	2 (11.8)	2 (22.2)	0 (0.0)	0.4706
**DS3 total, score**	16.8 ± 10.5	21.8 ± 5.6	11.9 ± 10.3	**0.0409**
**MSSI total, score**	18.9 ± 11.0	22.4 ± 10.5	15.4 ± 10.9	0.2133

Values are given as mean ± SD or median [range], if unequal distributed, or number (%). Platelet aggregation inhibitors include acetylsalicylic acid or clopidrogel. Anticoagulants include treatment with vitamin K antagonists (VKAs) or direct oral anticoagulants (DOACs/NOACs). Statistically significant differences between groups are shown in bold. ACR, albumin-creatinine ratio; ADA, anti-drug antibodies; DS3, Disease Severity Score System; eGFR, estimated glomerular filtration rate (CKD-EPI-based); ERT, enzyme replacement therapy; LVH, left ventricular hypertrophy defined as septum thickness >11.5 mm; lyso-Gb_3_, globotriaosylsphingosine (reference value ≤1.8 ng/ml); MSSI, Mainz Severity Score Index; RAAS, renin-angiotensin-aldosterone-system.

**Table 4:** Follow-up data of ERT-treated patients.

	total [n=17]	missense mutations [n=9]	nonsense mutations [n=8]	p-value
**age, years**	44.8 ± 19.0	52.9 ± 19.5	35.6 ± 14.7	0.0590
**follow-up, months**	25 [12 to 104]	25 [12 to 104]	26 [12 to 62]	0.7611
**lyso-Gb_3_, ng/ml**	28.6 [0.6 to 101.0]	19.0 [0.6 to 41.0]	34.6 [18.6 to 101.0]	**0.0464**
**agalsidase-alfa, n**	8 (47.1)	5 (55.6)	3 (37.5)	0.6372
**pre-medication, n**	10 (58.8)	4 (44.4)	6 (75.0)	0.3348
**lyso-lyso-Gb_3_ > reference, n**	16 (94.1)	8 (88.9)	8 (100.0)	0.9999
**neutralizing ADAs, n**	11 (64.7)	3 (33.3)	8 (100.0)	**0.0090**
**neutralizing capacity of ADAs, mg AGAL**	66 ± 48	31 ± 30	79 ± 48	0.1432
**serum creatinine, mg/dl**	0.85 [0.64 to 6.56]	1.50 [0.64 to 6.56]	0.81 [0.64 to 1.86]	0.2100
**eGFR, ml/min/1.73 m²**	85.4 ± 42.9	68.4 ± 43.9	104.5 ± 34.9	0.0828
**ACR, mg/g**	158 [0 to 3625]	1,679 [0 to 3625]	67 [5 to 1627]	0.5041
**albuminuria, n**	10 (62.5)	5 (62.5)	5 (62.5)	0.9999
**dialysis, n**	0 (0.0)	0 (0.0)	0 (0.0)	0.9999
**septum thickness, mm**	12.1 [8.6 to 22.0]	14.5 [9.0 to 21.0]	12.1 [8.6 to 22.0]	0.5506
**LVH, n**	11 (68.8)	6 (75.0)	5 (62.5)	0.9999
**myocardial infarction, n**	0 (0.0)	0 (0.0)	0 (0.0)	0.9999
**pacemaker/ICD, n**	6	4 (44.4)	2 (25.0)	0.6199
**stroke/TIA, n**	0 (0.0)	0 (0.0)	0 (0.0)	0.9999
**RAAS blockers, n**	9 (52.9)	7 (77.8)	2 (25.0)	0.0567
**diuretics, n**	2 (11.8)	2 (22.2)	0 (0.0)	0.4706
**DS3 total, score**	21 ± 14	25 ± 17	20 ± 11	0.2402
**MSSI total, score**	20 ± 11	22 ± 9	17 ± 12	0.3775

Pre-medication includes treatment with non-steroidal anti-inflammatory drug (NSAIDs), antihistamines, or glucocorticoids. Values are given as mean ± SD or median [range], if unequal distributed, or number (%). Statistically significant differences between groups are shown in bold. ACR, albumin-creatinine ratio; ADA, anti-drug antibodies; AGAL, α-galactosidase A; DS3, Disease Severity Score System; eGFR, estimated glomerular filtration rate (CKD-EPI-based); ERT, enzyme replacement therapy; ICD, implantable cardioverter defibrillator; LVH, left ventricular hypertrophy defined as septum thickness >11.5 mm; lyso-Gb_3_, globotriaosylsphingosine (reference value ≤1.8 ng/ml); MSSI, Mainz Severity Score Index; RAAS, renin-angiotensin-aldosterone-system.

Together, the clinical data demonstrate that treatment-naïve FD patients with nonsense mutations had significantly higher plasma lyso-Gb3 levels than patients with missense mutations. While ERT treatment strongly decreased lyso-Gb3 levels in both groups, they were still significantly higher in FD patients with nonsense mutations than in those with missense mutations. The higher lyso-Gb3 serum levels in FD patients with nonsense mutations were associated with a higher frequency of neutralizing ADAs in this group as compared with FD patients with missense mutations.

### 3.2 Complement activation in treatment-naive and ERT-treated FD patients

To assess potential complement activation in FD patient groups, we determined the concentration of small cleavage fragments of C3 and C5, i.e. the C3a and C5a anaphylatoxins, in the serum of treatment-naiüve FD patients and ERT-treatment.

**Figure 1:**
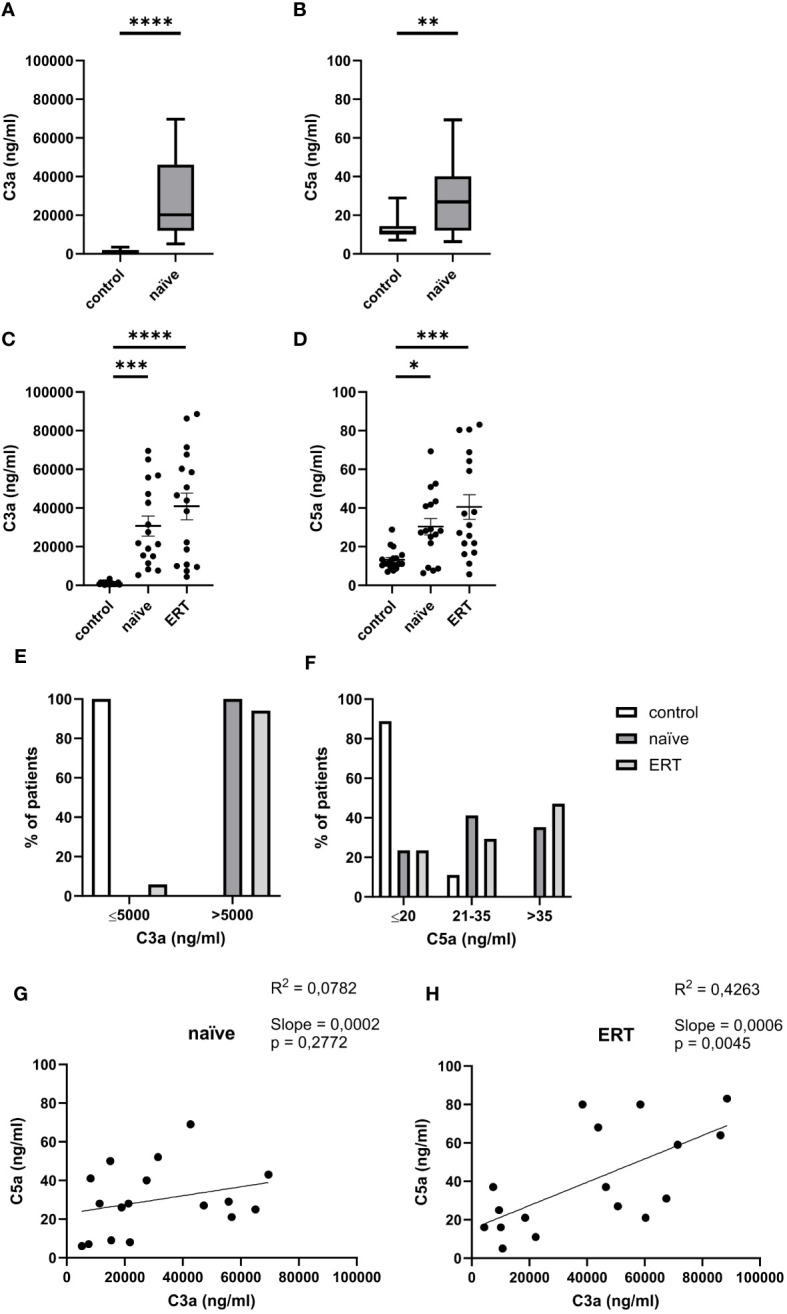
Serum concentrations of C3a and C5a in FD patients. **(A)** C3a and **(B)** C5a concentrations (ng/ml) in serum samples from healthy controls (n=18) and treatment-naiüve (n=24) FD patients. **(C)** C3a and **(D)** C5a concentrations (ng/ml) in serum samples from healthy controls (n=18), treatment-naiüve FD patients and from the same patients under ERT (n=17). Data were tested for normal distribution by Kolmogorov-Smirnov test. Differences between groups were determined by Mann-Whitney test **(A, B)** when data were not normally distributed or Ordinary one-way ANOVA with Tukey’s posthoc test **(C, D)** in case of normal distribution. Data in A, B are shown as median and range with 25th and 75th percentile. Data in **(C, D)** are shown as mean + SEM **(C, D)**. *p<0.05, **p<0.01; ***p<0.001; ****p<0.0001. **(E, F)** Percentage of serum samples from healthy controls (white bar, n=18), treatment-naiüve FD patients (black bar, n=17) and from the same patients under ERT (grey bar, n=17) with levels of **(E)** ≤5,000 ng/ml or >5,000 ng/ml of C3a or **(F)** ≤20 ng/ml, 21-35 ng/ml or >35 ng/ml C5a in serum. **(G, H)** Linear regression analysis of C5a and C3a serum levels (ng/ml) in FD patients before **(G)** and after **(H)** ERT (n=17). R^2^ is the coefficient of determination of linear regression and ranges from 0 (no linear regression) to 1 (perfect linear regression).

First, we measured C3a and C5a serum levels in 24 treatment-naive FD patients. Compared to healthy controls (935.8 ± 170.3 ng/ml), we detected 30-fold higher serum concentrations of C3a (27,620 ± 4,000 ng/ml) (**Figure 1A**) in these patients. Serum levels of C5a (28.0 ± 3.3 ng/ml) were twice as high as in healthy controls (13.2 ± 1.3) (**Figure 1B**). Next, we followed-up on 17 of the 24 FD patients and compared their C3a and C5a levels before and after the initiation of ERT. We not only found markedly increased C3a concentrations in FD patients before (30,660 ± 5,174 ng/ml) but also under ERT (40,872 ± 6,901 ng/ml) compared to healthy controls (**Figure 1C**). In addition, also C5a concentrations were significantly higher in treatment-naïve (30.4 ± 4.3 ng/ml) and ERT-treated FD patients (40.5 ± 6.4 ng/ml) as compared to healthy controls (**Figure 1D**). Thus, ERT was not associated with a reduction of the C3a and C5a concentrations. Given that age can affect complement activation (29), we performed linear regression analysis for C3a, C5a and age in the 17 follow-up patients (age range 20-76 years). However, we found no correlation between the anaphylatoxin serum levels and age (data not shown).

Of note, we found that the C3a serum concentrations of all subjects in the control group were ≤5,000 ng/ml, whereas C3a serum values in all treatment-naïve patients were >5,000 ng/ml. Also, only 6% of samples in the ERT-treated FD patient group showed C3a serum levels ≤5,000 ng/ml (**Figure 1E**). The C5a serum values were more heterogeneously distributed than the C3a serum levels in the FD group. As expected, most of the subjects in the control group (16 out of 18) had C5a serum levels ≤20 ng/ml. In treatment-naïve FD patients, we found three different groups: (i) those with C5a values ≤20 ng/ml (n=6 (25%)); (ii) between 21 - 35 ng/ml (n=11 (46%)), and (iii) > 35 ng/ml (n=7 (29%)). Out of the 17 patients investigated under ERT, four had C5a serum values ≤20 ng/m (24%); five between 21 - 35 ng/ml (29%) and eight C5a values >35 ng/ml (47%) suggesting higher complement activation in the course of the disease which was not affected by ERT (**Figure 1F**).

Linear regression analysis between serum C3a and C5a levels showed no significant correlation in treatment-naïve patients (**Figure 1G**). In contrast, we found a significant positive correlation between C3a and C5a serum concentrations under ERT treatment (**Figure 1H**). Of note, the disease severity assessed as total MSSI and DS3 scores did not correlate with levels of the anaphylatoxins C3a and C5a before or under ERT (data not shown).

### 3.3 Increased complement activation in Fabry disease patients with nonsense mutations despite ERT

**Figure 2:**
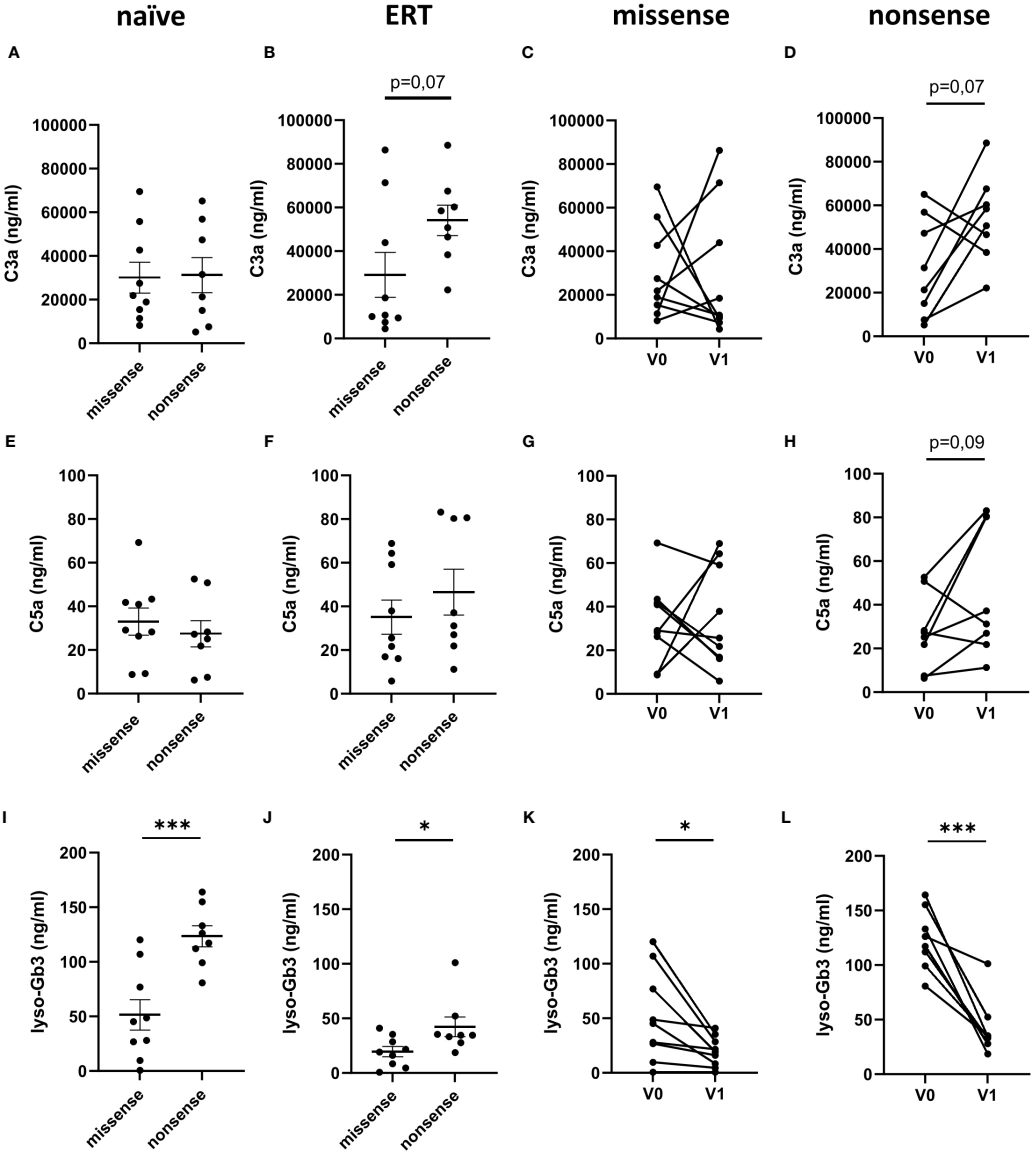
C3a, C5a and lyso-Gb3 concentrations in the circulation of FD patients with missense/nonsense mutations. **(A–D)** C3a, **(E–H)** C5a and **(I–L)** lyso-Gb3 concentrations (ng/ml) in samples from **(A, E, I)** treatment-naiüve (n=17), **(B, F, J)** FD patients under ERT (n=17) with missense (n=9) or nonsense (n=8) *GLA* mutations and from FD patients with **(C, G, K)** missense (n=9) or **(D, H, L)** nonsense (n=8) *GLA* mutations before (V0) or under ERT (V1). Data were tested for normal distribution by Kolmogorov-Smirnov test. Differences between groups were determined by unpaired t-test **(A, B, E, F, I, J**) or paired t- test **(C, D, G, H, K, L)**. Data in **(A, B, E, F, I, J)** are shown as mean + SEM. *p<0.05; ***p<0.001.

In the next step, we sought to determine a potential impact of the *GAL* gene mutations on complement activation in FD patients. For this purpose, we compared serum C3a and C5a levels in FD patients with either nonsense or missense mutations before and under ERT. Anaphylatoxin concentrations were comparable in FD patients with missense or nonsense mutations before ERT initiation (**Figures 2A, E**). In contrast, we observed a trend toward increased C3a (54,085 ± 7,017 ng/ml; p=0.07) but not C5a (46.5 ± 10.5 ng/ml) serum levels increased in FD patients with nonsense mutations as compared to those with missense mutations (C3a: 29,126 ± 10,245 ng/ml; C5a: 35.1 ± 7.8 ng/ml) (**Figures 2B, C, G, F**). As expected, plasma lyso-Gb3 levels were significantly higher in treatment-naïve FD patients with nonsense mutations (123.4 ± 9.8 ng/ml) than in those with missense mutations (51.4 ± 13.9 ng/ml) (**Figure 2I**). Even after ERT initiation, the lyso-Gb3 levels were still significantly higher (42.2 ± 9.0 ng/ml) in the nonsense than in the missense mutation group (19.4 ± 4.6 ng/ml) (**Figure 2J**).

When we compared the C3a and C5a serum levels in FD patients with missense or nonsense mutations before and under ERT, we found increased C3a and the C5a levels after ERT initiation in six of the eight FD patients with nonsense mutations (**Figures 2D, H**). In contrast, we did not see this dominant increase in the group of FD patients with missense mutations. As expected, lyso-Gb3 levels significantly declined in FD patients with missense or nonsense mutations after ERT initiation (**Figures 2K, L**). Thus, while ERT treatment was able to compensate in large part the enzyme defect, it had no impact on the activation of the complement system, which was even upregulated under ERT in FD patients with nonsense mutations. Of note, also the disease burden, as determined by the MSSI and DS3 scores, did not correlate with lyso-Gb3 serum concentrations in naïve or ERT-treated FD patients (data not shown).

### 3.4 Increased complement activation in ADA-positive Fabry disease patients under ERT

**Figure 3:**
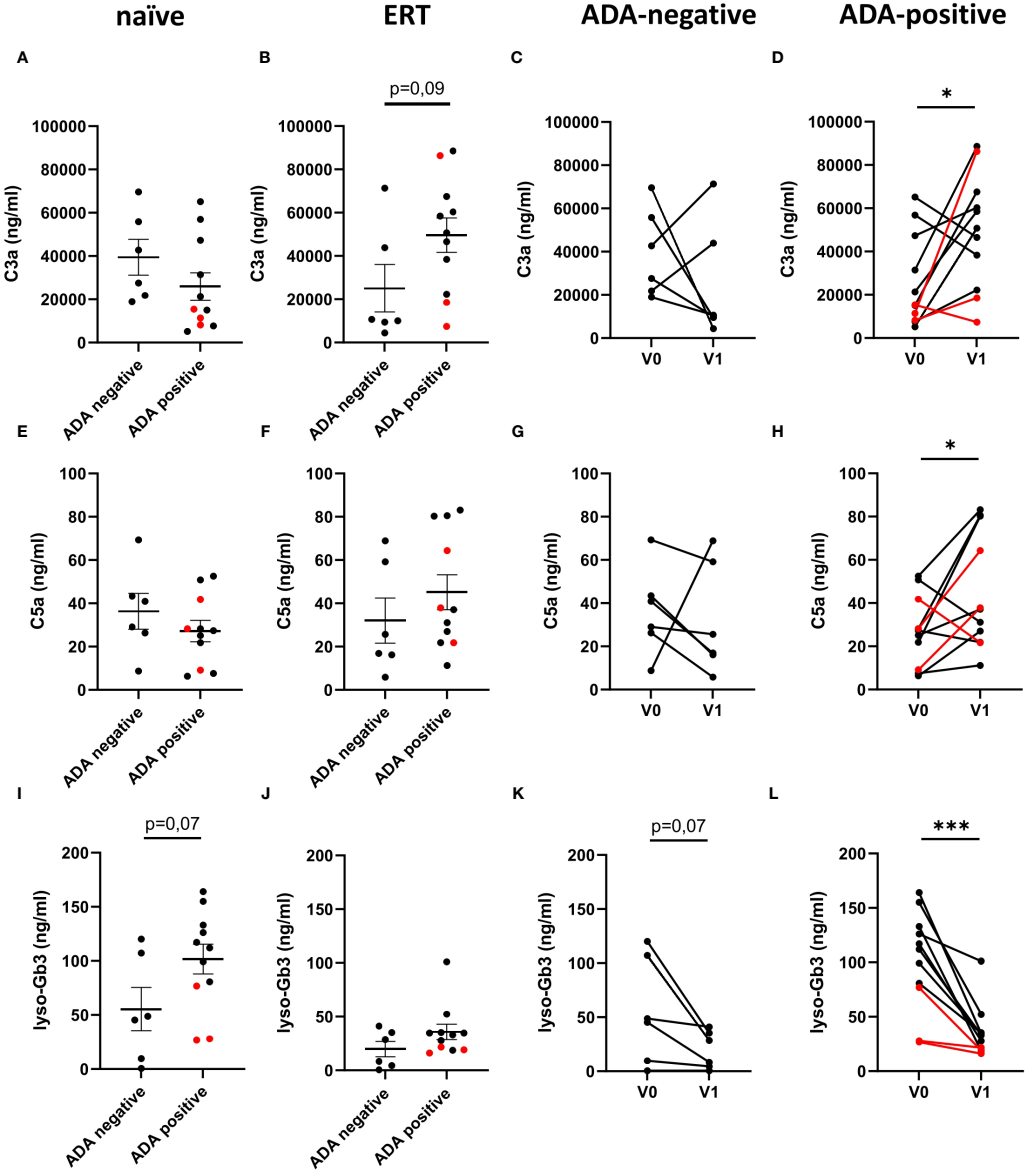
C3a, C5a and lyso-Gb3 concentrations in the circulation of FD patients with ADA-negative/positive status. **(A–D)** C3a, **(E–H)** C5a and **(I–L)** lyso-Gb3 concentrations (ng/ml) in serum from ADA-negative (n=6) or ADA-positive (n=11) **(A, E, I)** patients, who were either treatment-naiüve (n=17) or **(B, F, J)** received ERT (n=17). **(C, G, K)** ADA-negative (n=6) or **(D, H, L)** ADA-positive (n=11) FD patients before (V0) or under ERT (V1). Red lines denote patients with missense mutations. Data were tested for normal distribution by Kolmogorov-Smirnov test. Differences between groups were determined by unpaired t-test **(A, B, E, F, I, J)** or paired t-test **(C, D, G, H, K, L)**. Data in **(A, B, E, F, I, J)** are shown as mean + SEM. *p<0.05; ***p<0.001.

Finally, we assessed if the presence of neutralizing ADAs affects complement activation in FD patients. We compared serum C3a and C5a levels in ADA-negative or ADA-positive FD patients before and under ERT. C3a and C5a serum levels were comparable in ADA-negative and ADA-positive FD patients before ERT initiation (**Figures 3A, E**). Under ERT, ADA-positive FD patients showed a tendency toward higher C3a levels (49,538 ± 7,982 ng/ml; p=0.09) (**Figure 3B**) but not C5a levels (45.1 ± 8.1 ng/ml) (**Figure 3F**) than ADA negative FD patients (C3a: 24,984 ± 10,960 ng/ml; C5a: 32.1 ± 10.5 ng/ml) (**Figures 3C, G**). Lyso-Gb3 levels were somewhat higher in ADA-positive (101.6 ± 13.7 ng/ml) than in ADA-negative (55.2 ± 20.1 ng/ml) FD patients before (**Figure 3I**: p=0.07) but not after ERT initiation (35.8 ± 7.2 ng/ml vs. 19.7 ± 7.1 ng/ml) (**Figure 3J**).

Comparing C3a and C5a serum concentrations before and under ERT, we found significantly increased C3a (**Figure 3D**) and C5a (**Figure 3H**) concentrations in ADA-positive patients, whereas anaphylatoxin levels were heterogenous in ADA-negative FD patients after ERT. In contrast to the increased C3a and C5a levels in ADA-positive patients, their lyso-Gb3 levels significantly decreased (**Figure 3L**). A decrease was also evident in ADA-negative FD patients under ERT (**Figure 3K**; p=0.07), albeit to a lesser degree.

Together, these finding demonstrate that despite successful reduction of lyso-Gb3 levels by ERT, complement activation still proceeds and is even higher under ERT in FD diseases patients that developed ADAs.

### 3.5 Low pro-inflammatory cytokine and chemokine response in FD patients but increased anti-inflammatory response after ERT therapy

**Figure 4:**
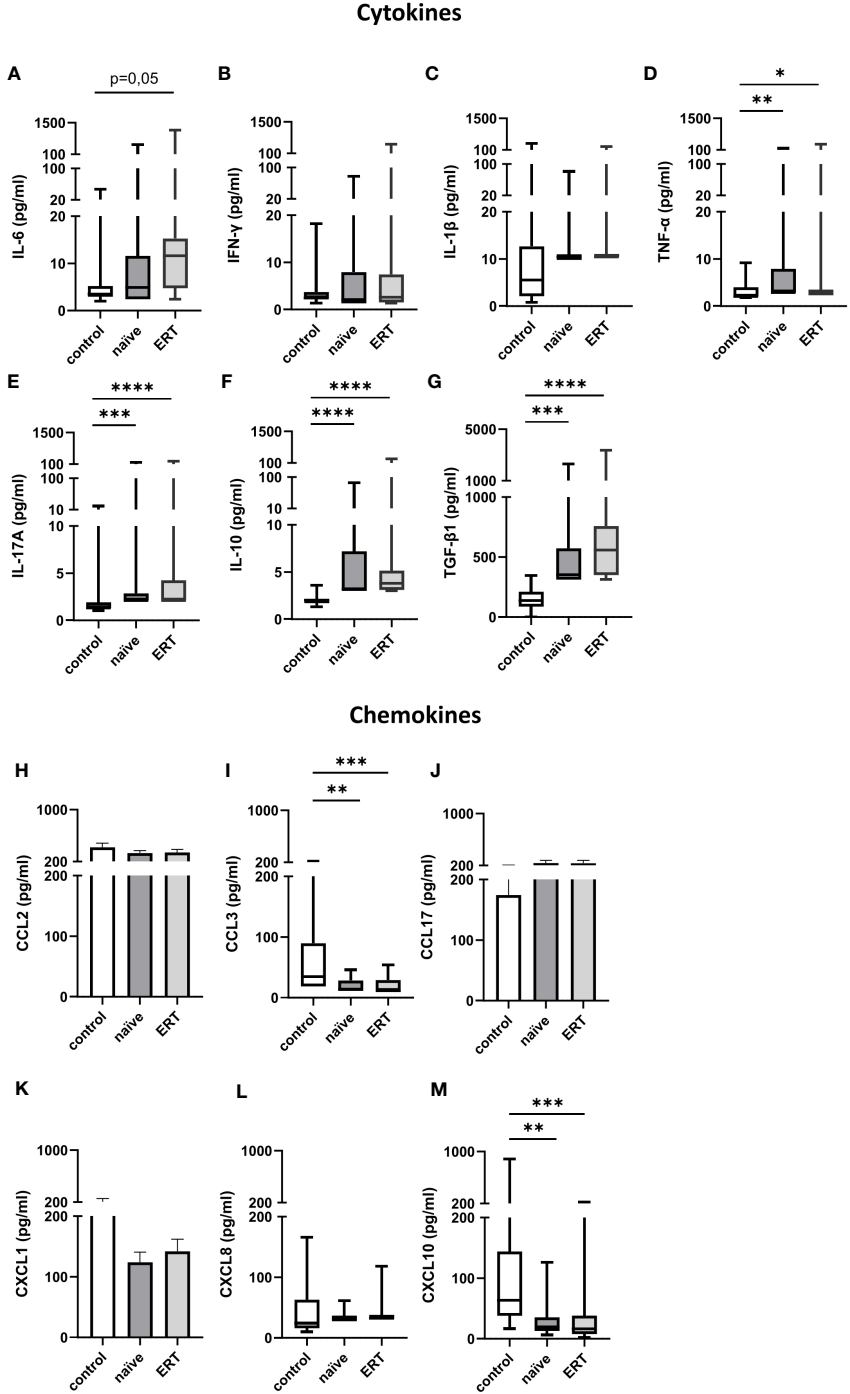
Cytokine and chemokine serum concentrations in FD patients before and under ERT. **(A)** IL-6, **(B)** IFN-γ, **(C)** IL-1β, **(D)** TNF-α, **(E)** IL-17A, **(F)** IL-10 and **(G)** TGF-β1 as well as **(H)** CCL2, **(I)** CCL3, **(J)** CCL17, **(K)** CXCL1 **(L)** CXCL8 and **(M)** CXCL10 concentrations (pg/ml) in serum samples from healthy controls (n=18), and FD patients before and under ERT-treatment (n=17). Cytokine and chemokine concentrations were determined by LEGENDplex™. Data were tested for normal distribution by Kolmogorov-Smirnov test. Differences between groups were determined by Kruskal-Wallis test with Dunn’s posthoc test **(A-G, I, L, M)** or Ordinary one-way ANOVA with Tukey’s posthoc test **(H, J, K)**. Data in **(A–G, I, L, M)** are shown as median and range with 25th and 75th percentile. Data in **(H, J, K)** are shown as mean + SEM. *p<0.05; **p<0.01; ***p<0.001; ****p<0.0001.

In addition to the humoral inflammatory response, we determined immune cell activation in FD patients. First, we assessed the concentrations of pro- (IL-6, IFN-γ, IL-1β, TNF-α and IL-17A) (**Figures 4A–E**) and anti-inflammatory (IL-10 and TGF-β1) (**Figures 4F, G**) cytokines as well as the levels of CCL (CCL2/MCP-1, CCL3/MIP-1α and CCL17/TARC) (**Figures 4H–J**) and CXCL (CXCL1/GROα, CXCL8/IL-8 and CXCL10/IP-10) (**Figures 4K–M**) chemokines in the serum of treatment-naiüve patients and under ERT. Except TGF-β1, serum levels of all other cytokines were very low in healthy controls and in FD patients independent of ERT. Serum levels of TNF-α (2.9 [2.9-164.2] pg/ml; median [range]), IL-17A (2.1 [2.1-177.6] pg/ml), IL-10 (3.2 [3.0-84.0] pg/ml) and TGF-β1 (351.6 [312.0-2,295] pg/ml) were significantly increased in treatment-naive patients as compared with healthy controls (TNF-α: 2.0 [1.8-9.2] pg/ml; IL-17A: 1.3 [1.0-20.1] pg/ml; IL-10: 2.0 [1.3-3.6] pg/ml; TGF-β1 (137.2 [1.0-344.7] pg/ml) (**Figures 4D–G**).

After ERT initiation, serum level of TNF-α (2.9 [2.9-348.4] pg/ml), IL-17A (2.1 [2.1-221.6] pg/ml), IL-10 (3.8 [3.0-283.5] pg/ml) and TGF-β1 (557.8 [312.0-3,358] pg/ml) (**Figures 4D–G**) remained significantly increased as compared to healthy controls. Further, we observed a trend toward increased serum concentration of IL-6 as compared to healthy controls (11.6 [2.4-1,168] pg/ml vs. 3.5 [2.0-46.5] pg/ml, p=0.05).

Surprisingly, serum levels of macrophage/monocyte derived CCL3/MIP-1α and CXCL10/IP-10 were significantly decreased in treatment-naïve FD patients (CCL3/MIP-1α: 13.7 [11.2-45.9] pg/ml; CXCL10/IP-10: 19.5 [6.3-126.1] pg/ml) and after ERT initiation (CCL3/MIP-1α: 11.2 [11.2-54.0] pg/ml; CXCL10/IP-10: 16.2 [1.9-217.4] pg/ml) (**Figures 4I, M**) as compared to the control group of healthy subjects (CCL3/MIP-1α: 34.5 [18.6-218.1] pg/ml; CXCL10/IP-10: 63.4 [16.6-885.1] pg/ml). In contrast, CCL2, CCL17, CXCL1 and CXCL8 serum concentrations were similar in healthy individuals and FD patients independent of ERT.

### 3.6 Increased cytokine production in ERT-treated FD patients with missense mutations and in the absence of ADAs

**Figure 5:**
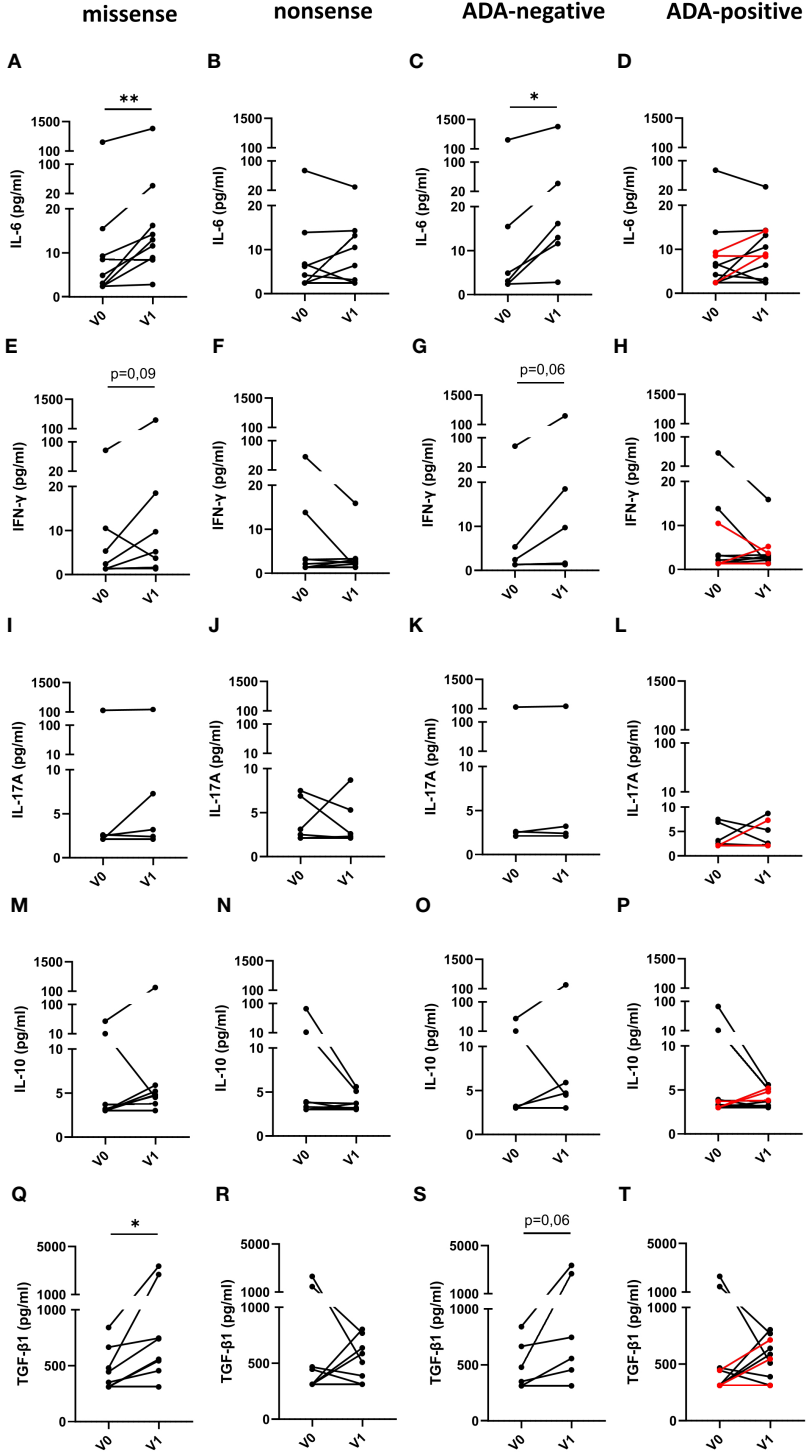
Impact of the mutation status and the generation of ADAs on serum cytokine serum concentrations in FD patients before and under ERT. **(A–D)** IL-6, **(E–H)** IFN-γ, **(I–L)** IL-17A, **(M–P)** IL-10 and **(Q–T)** TGF-β1 concentrations (pg/ml) in serum samples from FD patients with **(A, E, I, M, Q)** missense (n=9) or **(B, F, J, N, R)** nonsense (n=8) *GLA* mutations in **(C, G, K, O, S)** and ADA-negative (n=6) or **(D, H, L, P, T)** ADA-positive (n=11) FD patients before (V0) or under ERT (V1). Red lines denote patients with missense mutation. Data were tested for normal distribution by Kolmogorov-Smirnov test. Differences between groups were determined by Wilcoxon matched-pairs signed rank test. *p<0.05; ** p<0.01.

Subsequently, we determined, if the type of mutation or the presence or absence of neutralizing ADAs in FD patients has any impact on immune cell activation (**Figures 5A–T**). For this purpose, we compared serum cytokine and chemokine levels of FD patients with missense or nonsense mutations as well as with ADAs or without ADAs before and under ERT. Intriguingly, IL-6 (**Figure 5A**) and TGF-β1 (**Figure 5Q**) serum levels in ERT-treated FD patients with missense but not with nonsense mutations significantly increased as compared with cytokine serum concentrations before ERT started. Further, we found a trend toward increased IFN-γ serum levels (**Figure 5E**) in FD patients with missense mutations under ERT (p=0.09). Also, IL-6 (**Figure 5C**) serum levels were significantly increased and IFN-γ (**Figure 5G**) as well as TGF-β1 (**Figure 5S**) levels were higher in most ADA-negative but not in ADA-positive FD patients under ERT (p=0.06).

**Figure 6:**
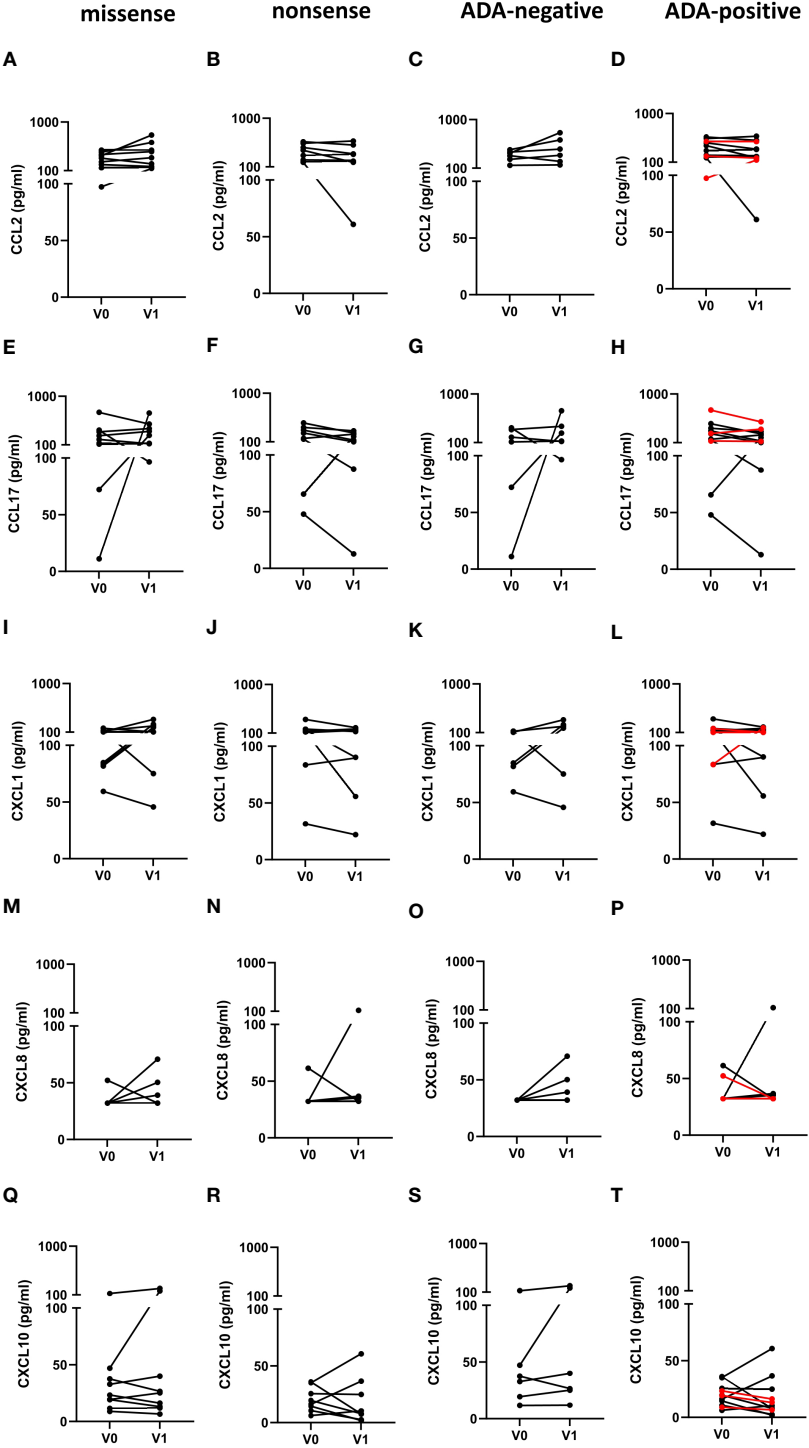
Impact of the mutation status and the generation of ADAs on serum chemokine serum concentrations in FD patients before and under ERT. **(A–D)** CCL2, **(E–H)** CCL17, **(I–L)** CXCL1, **(M–P)** CXCL8 and **(Q–T)** CXCL10 concentrations (pg/ml) in serum samples from FD patients with **(A, E, I, M, Q)** missense (n=9) or **(B, F, J, N, R)** nonsense (n=8) *GLA* mutations in **(C, G, K, O, S)** ADA-negative (n=6) or **(D, H, L, P, T)** ADA-positive (n=11) FD patients before (V0) or under ERT (V1). Red lines denote patients with missense mutations. Data were tested for normal distribution by Kolmogorov-Smirnov test. Differences between groups were determined by paired t-test **(A–L, Q–T)** or Wilcoxon matched-pairs signed rank test **(M–P)**.

Finally, we determined, if the type of mutation or the ADA status in FD patients affected the chemokine serum levels in response to ERT. However, in contrast to the cytokine serum levels, we found no impact of the different mutations or the ADA status on the chemokine response after ERT (**Figures 6A–T**).

## 4 Discussion

In this study, we identified a hitherto unrecognized strong complement activation in treatment-naive classic male FD patients with missense or nonsense mutations as evidenced by high serum levels of the anaphylatoxins C3a and C5a, the small cleavage products of C3 and C5. Previously, increased levels of C1q, C3 and C4 have been observed in the circulation by proteomic analysis in treatment-naïve male FD patients as compared with control individuals. They decreased under ERT, which pointed toward the activation of the system (30). However, direct activation of the complement system has not been assessed in this study. In line, we found 30-fold increased C3a serum levels in adult FD patients when compared to those of healthy controls. In addition, C5a levels were significantly higher in such FD patients than in healthy individuals, although they increased only 2-fold. This difference is most likely due to the different biologic properties of the primary degradation products of C3a and C5a, i.e. C3adesArg and C5adesArg, which quickly emerge in response to serum carboxypeptidase N, cleaving-off the C-terminal arginine from the two anaphylatoxins (31). Importantly, C3adesArg is biologically inert, whereas C5adesArg still binds with high affinity to its receptors C5aR1 and C5aR2, present on many circulating immune cells (31). As age can affect complement activation (29), we also determined a potential correlation between anaphylatoxin serum levels and age. While we did not find such a correlation, we cannot exclude that complement activation is different in children and adolescent FD patients. The complement activation, i.e. the serum C3a concentrations that we observed in the FD patients are not only much higher than in healthy controls but markedly higher than the C3a levels determined in two immune-complex mediated diseases, in which complement activation has previously been demonstrated, i.e. systemic lupus erythematosus (32) or anti-phosphlipd syndrome (33) and similar to C3a levels observed in anti-neutrophil cytoplasmic antibody (ANCA)-associated vasculits (34).

Intriguingly, we found that all treatment-naïve FD patients but none of the healthy subjects had C3a serum levels >5,000 ng/ml. Even after ERT, C3a levels of almost all FD patients remained above this value, whereas the C5a serum pattern was more complex before and after ERT initiation. These data suggest strong complement activation at the level of C3 in FD patients independent of lyso-Gb3 levels and identify complement activation as potential new mechanisms contributing to the inflammatory response in FD. In support of lyso-Gb3-independent complement activation, we found significantly higher C3a and C5a levels after ERT in ADA-positive patients, comprising all patients with nonsense and three patients with missense mutations.

In most FD patients with nephropathy, no glomerular deposition of immunoglobulins or complement cleavage products have been found (16). However, some C3b deposition in the glomerular mesangial area has been described in 8 out of 11 FD patients suffering from glomerulopathy (30). Also, in a patient with Pompe disease, who developed secondary membranous nephropathy in response to ERT, subepithelial C3 and C5b-9 deposits have been observed (35). Importantly, numerous studies provide evidence that complement activation by the classical, lectin and alternative pathway serves as a critical driver of the immunopathology in common glomerular kidney diseases such as IgA nephropathy (36) and lupus nephritis (37) or rare diseases including C3 glomerulopathy (38), atypical hemolytic uremic syndrome (aHUS) (39) as well as ANCA-associated vasculits (40). Thus, complement may also play an important role as a driver of Fabry nephropathy. Preventing the conversion from C5 to C5a and C5b using C5-blocking antibodies such as eculizumab have been successfully used in aHUS (39). Also, targeting the C5a/C5aR1 using the small molecule inhibitor avacopan has been approved for ANCA-associated vasculitis and is now frequently used in the clinic (41). While cytokines, epithelial-mesenchymal trans-differentiation, oxidative stress and remodeling of vascular cells have been identified to promote ongoing kidney inflammation and, eventually, kidney fibrosis in response to Gb3 (16), a potential role of complement remains to be explored. Clearly, complement-mediated activation of myeloid cells contributes to renal fibrosis in chronic kidney diseases (42, 43).

Similar to the strong complement activation in FD patients, we previously observed strong complement activation in clinical and experimental GD, which was associated with high serum levels of IgG auto-antibodies directed against glucosylceramide as a driver of complement activation by the classical pathway (22). Our observation that C3a serum levels were higher in ADA-positive than in ADA-negative patients may point toward complement activation by the classical and/or the lectin pathway via ADA immune complexes. ADAs are mainly composed of IgG1 and IgG4 antibody subtypes (44). While IgG1 antibodies can activate the complement system by the classical pathway, it was recently demonstrated that aberrantly glycosylated IgG4, lacking Fc glycan galactosylation, can activate complement by the lectin pathway (45). Further, such aberrant glycosylation of IgG Fc glycans increases the pro-inflammatory potential of such antibodies (46, 47). It will be interesting to determine, if such aberrant glycosylation pattern also occurs in FD. Of note, the presence of ADAs has not been associated with glomerular IgG deposition and drug-associated membraneous nephropathy as has been described for other drugs such as penicillamine or captopril among others (48). In addition to ADA immune complexes, other mechanisms contribute to the complement activation in FD, as we also detected strong complement activation in patients with missense mutations lacking ADAs and in treatment-naïve FD patients. Potential mechanisms include increased cellular complement production and activation due to the massive Gb3 accumulation in immune cells. Indeed, in experimental GD, we found increased C5 production and C5a generation in macrophages and dendritic cells, adding to the immune complex-mediated activation of complement (22). Mechanistically, intracellular canonical complement activation by one of the three pathways as well as non-canonical activation by cellular proteases may account for such activation. Intracellular production of Factors B and D has been described together with C3 and C5 production which could result in AP activation. Alternatively, and not mutually exclusive, proteases such as cathepsin L may cleave C3 into C3a and C3b (49). Strikingly, activation of the C5a/C5aR1 axis in Gaucher cells increased expression of UDP-glucose ceramide glucosyltransferase (GCS), which may also tip the balance toward more production of lactosylceramide and, eventually, fuel intracellular Gb3 accumulation in FD.

Chronic inflammation due to continuous activation of innate immune cells such as neutrophils, monocytes and macrophages has been suggested to contribute to organ damage in FD (15, 16, 50). Among the tested cytokines, pro- (IL-6, TNF-α and IL-17) and anti- (IL-10 and TGF-ß1) inflammatory cytokines were elevated in FD patients, in particular in patients with missense mutations after ERT. In contrast, IFN-γ and IL-1ß serum concentrations were indistinguishable from healthy controls subjects. Importantly, FD patients with missense mutations suffered from early-stage kidney disease as indicated by a reduced eGFR associated with increased serum creatinine levels. Several kidney cells including podocytes and mesangial cells can produce IL-6, TGF-ß1 and IL-10 and elevated levels of such cytokines have been demonstrated in several chronic kidney diseases (51–53). Indeed, high TGF-ß1 production by proximal tubular cells has been reported in renal biopsies of FD patients suggesting production of inflammatory and pro-fibrotic cytokines from kidney cells of FD patients (54). Elevated TGF-ß levels have previously been shown to be associated with lower eGFR and an increased risk of cardiovascular events (55). It remains to be determined, if the increased levels of TGF-ß that we observed in patients with missense mutations may serve as biomarker for risk assessment of myocardial infarction or stroke, which frequently occurs in FD patients.

In contrast to the increased serum concentrations of some cytokines, one CCL (CCL3) and one CXCL chemokine (CXCL10) were reduced in FD patients both before and after ERT. At this point, potential mechanisms and consequences are unclear.

In summary, we identified strong complement activation in FD patients, which proceeded independent of lyso-Gb3 reduction after ERT. Such complement activation may drive the immunopathology of FD at several levels: (i) through its impact on glucosphingolipid metabolism (56); (ii) cross-talk with Toll-like receptors (57, 58); and (iii) proinflammatory cytokine induction in the kidney (59), which could fuel profibrotic pathways induced by TGF-ß1, IL-6 and IL-10. At this point, it remains to be determined which mechanisms are the main drivers of complement activation. Whatever the mechanisms are, complement targeting might prove useful as an additional therapeutic option to reduce the ongoing humoral and cellular inflammation that seems to drive disease progression despite ERT. Several complement inhibitors have been approved and are available that target the complement system at the level of C1 (60), C3 (61), C5 (62) or C5aR1 (63).

## Data availability statement

The original contributions presented in the study are included in the article/supplementary material. Further inquiries can be directed to the corresponding authors.

## Ethics statement

The studies involving humans were approved by Medical Association of Westphalian-Lippe and Ethics Committee of the Medical Faculty of the University of Muenster and Ethics Committee of the University of Lübeck. The studies were conducted in accordance with the local legislation and institutional requirements. The participants provided their written informed consent to participate in this study.

## Author contributions

BL: Investigation, Project administration, Writing – original draft, Writing – review & editing. ML: Investigation, Resources, Writing – review & editing. EE-J: Investigation, Writing – review & editing. KH: Conceptualization, Writing – review & editing. EB: Conceptualization, Resources, Supervision, Writing – review & editing. JK: Conceptualization, Funding acquisition, Project administration, Supervision, Writing – original draft, Writing – review & editing.

## References

[1] Zarate YA Hopkin RJ. Fabry's disease. Lancet (2008) 372:1427–35. doi: 10.1016/S0140-6736(08)61589-5 18940466

[2] Desnick RJ Wasserstein MP. Fabry disease: clinical features and recent advances in enzyme replacement therapy. Adv Nephrol Necker Hosp (2001) 31:317–39. 11692469

[3] Germain DP. Fabry disease. Orphanet J Rare Dis (2010) 5:30. doi: 10.1186/1750-1172-5-30 21092187 PMC3009617

[4] Garman SC. Structure-function relationships in alpha-galactosidase A. Acta Paediatr (2007) 96:6–16. doi: 10.1111/j.1651-2227.2007.00198.x PMC3065945 17391432

[5] Ranieri M Bedini G Parati EA Bersano A. Fabry disease: recognition, diagnosis, and treatment of neurological features. Curr Treat Options Neurol (2016) 18:33. doi: 10.1007/s11940-016-0414-5 27225543

[6] Mort M Ivanov D Cooper DN Chuzhanova NA. A meta-analysis of nonsense mutations causing human genetic disease. Hum Mutat (2008) 29:1037–47. doi: 10.1002/humu.20763 18454449

[7] Romeo G D'urso M Pisacane A Blum E De Falco A Ruffilli A. Residual activity of alpha-galactosidase A in Fabry's disease. Biochem Genet (1975) 13:615–28. doi: 10.1007/BF00484919 812485

[8] Yam GH Zuber C Roth J. A synthetic chaperone corrects the trafficking defect and disease phenotype in a protein misfolding disorder. FASEB J (2005) 19:12–8. doi: 10.1096/fj.04-2375com 15629890

[9] Lenders M Brand E. Fabry disease: the current treatment landscape. Drugs (2021) 81:635–45. doi: 10.1007/s40265-021-01486-1 PMC8102455 33721270

[10] Eng CM Guffon N Wilcox WR Germain DP Lee P Waldek S. Safety and efficacy of recombinant human alpha-galactosidase A replacement therapy in Fabry's disease. N Engl J Med (2001) 345:9–16. doi: 10.1056/NEJM200107053450102 11439963

[11] Schiffmann R Kopp JB Austin HA 3rd Sabnis S Moore DF Weibel T. Enzyme replacement therapy in Fabry disease: a randomized controlled trial. JAMA (2001) 285:2743–9. doi: 10.1001/jama.285.21.2743 11386930

[12] Germain DP Hughes DA Nicholls K Bichet DG Giugliani R Wilcox WR. Treatment of fabry's disease with the pharmacologic chaperone migalastat. N Engl J Med (2016) 375:545–55. doi: 10.1056/NEJMoa1510198 27509102

[13] Hughes DA Nicholls K Shankar SP Sunder-Plassmann G Koeller D Nedd K. Oral pharmacological chaperone migalastat compared with enzyme replacement therapy in Fabry disease: 18-month results from the randomised phase III ATTRACT study. J Med Genet (2017) 54:288–96. doi: 10.1136/jmedgenet-2016-104178 PMC5502308 27834756

[14] Lenders M Brand E. Effects of enzyme replacement therapy and antidrug antibodies in patients with fabry disease. J Am Soc Nephrol (2018) 29:2265–78. doi: 10.1681/ASN.2018030329 PMC6115664 30093456

[15] De Francesco PN Mucci JM Ceci R Fossati CA Rozenfeld PA. Fabry disease peripheral blood immune cells release inflammatory cytokines: role of globotriaosylceramide. Mol Genet Metab (2013) 109:93–9. doi: 10.1016/j.ymgme.2013.02.003 23452955

[16] Feriozzi S Rozenfeld P. Pathology and pathogenic pathways in fabry nephropathy. Clin Exp Nephrol (2021) 25:925–34. doi: 10.1007/s10157-021-02058-z 33768330

[17] Jehn U Bayraktar S Pollmann S Van Marck V Weide T Pavenstadt H. alpha-galactosidase a deficiency in fabry disease leads to extensive dysregulated cellular signaling pathways in human podocytes. Int J Mol Sci (2021) 22:11339. doi: 10.3390/ijms222111339 PMC8583658 34768768

[18] Linthorst GE Hollak CE Donker-Koopman WE Strijland A Aerts JM. Enzyme therapy for Fabry disease: neutralizing antibodies toward agalsidase alpha and beta. Kidney Int (2004) 66:1589–95. doi: 10.1111/j.1523-1755.2004.00924.x 15458455

[19] Vedder AC Breunig F Donker-Koopman WE Mills K Young E Winchester B. Treatment of Fabry disease with different dosing regimens of agalsidase: effects on antibody formation and GL-3. Mol Genet Metab (2008) 94:319–25. doi: 10.1016/j.ymgme.2008.03.003 18424138

[20] Rombach SM Aerts JM Poorthuis BJ Groener JE Donker-Koopman W Hendriks E. Long-term effect of antibodies against infused alpha-galactosidase A in Fabry disease on plasma and urinary (lyso)Gb3 reduction and treatment outcome. PloS One (2012) 7:e47805. doi: 10.1371/journal.pone.0047805 23094092 PMC3477102

[21] Lenders M Stypmann J Duning T Schmitz B Brand SM Brand E. Serum-mediated inhibition of enzyme replacement therapy in fabry disease. J Am Soc Nephrol (2016) 27:256–64. doi: 10.1681/ASN.2014121226 PMC4696578 25933799

[22] Pandey MK Burrow TA Rani R Martin LJ Witte D Setchell KD. Complement drives glucosylceramide accumulation and tissue inflammation in Gaucher disease. Nature (2017) 543:108–12. doi: 10.1038/nature21368 28225753

[23] Levey AS Stevens LA Schmid CH Zhang YL Castro AF 3rd Feldman HI. A new equation to estimate glomerular filtration rate. Ann Intern Med (2009) 150:604–12. doi: 10.7326/0003-4819-150-9-200905050-00006 PMC2763564 19414839

[24] Williams B Mancia G Spiering W Agabiti Rosei E Azizi M Burnier M. 2018 ESC/ESH Guidelines for the management of arterial hypertension. Eur Heart J (2018) 39:3021–104. doi: 10.1093/eurheartj/ehy339 30165516

[25] Giannini EH Mehta AB Hilz MJ Beck M Bichet DG Brady RO. A validated disease severity scoring system for Fabry disease. Mol Genet Metab (2010) 99:283–90. doi: 10.1016/j.ymgme.2009.10.178 19951842

[26] Whybra C Kampmann C Krummenauer F Ries M Mengel E Miebach E. The Mainz Severity Score Index: a new instrument for quantifying the Anderson-Fabry disease phenotype, and the response of patients to enzyme replacement therapy. Clin Genet (2004) 65:299–307. doi: 10.1111/j.1399-0004.2004.00219.x 15025723

[27] Lenders M Schmitz B Brand SM Foell D Brand E. Characterization of drug-neutralizing antibodies in patients with Fabry disease during infusion. J Allergy Clin Immunol (2018) 141:2289–2292.e2287. doi: 10.1016/j.jaci.2017.12.1001 29421273

[28] Fenton A Montgomery E Nightingale P Peters AM Sheerin N Wroe AC. Glomerular filtration rate: new age- and gender- specific reference ranges and thresholds for living kidney donation. BMC Nephrol (2018) 19:336. doi: 10.1186/s12882-018-1126-8 30466393 PMC6249883

[29] Gaya Da Costa M Poppelaars F Van Kooten C Mollnes TE Tedesco F Wurzner R. Age and sex-associated changes of complement activity and complement levels in a healthy caucasian population. Front Immunol (2018) 9:2664. doi: 10.3389/fimmu.2018.02664 30515158 PMC6255829

[30] Heo SH Kang E Kim YM Go H Kim KY Jung JY. Fabry disease: characterisation of the plasma proteome pre- and post-enzyme replacement therapy. J Med Genet (2017) 54:771–80. doi: 10.1136/jmedgenet-2017-104704 PMC5740533 28835480

[31] Klos A Tenner AJ Johswich KO Ager RR Reis ES Kohl J. The role of the anaphylatoxins in health and disease. Mol Immunol (2009) 46:2753–66. doi: 10.1016/j.molimm.2009.04.027 PMC2725201 19477527

[32] Cai YH Deng J Chen ZL Mei H Tang L Luo SS. Brief report on the relation between complement C3a and anti dsDNA antibody in systemic lupus erythematosus. Sci Rep (2022) 12:7098. doi: 10.1038/s41598-022-10936-z 35501405 PMC9061720

[33] Oku K Amengual O Hisada R Ohmura K Nakagawa I Watanabe T. Autoantibodies against a complement component 1 q subcomponent contribute to complement activation and recurrent thrombosis/pregnancy morbidity in anti-phospholipid syndrome. Rheumatol (Oxford) (2016) 55:1403–11. doi: 10.1093/rheumatology/kew196 27084309

[34] Moiseev S Lee JM Zykova A Bulanov N Novikov P Gitel E. The alternative complement pathway in ANCA-associated vasculitis: further evidence and a meta-analysis. Clin Exp Immunol (2020) 202:394–402. doi: 10.1111/cei.13498 32691878 PMC7670131

[35] Debiec H Valayannopoulos V Boyer O Noel LH Callard P Sarda H. Allo-immune membranous nephropathy and recombinant aryl sulfatase replacement therapy: a need for tolerance induction therapy. J Am Soc Nephrol (2014) 25:675–80. doi: 10.1681/ASN.2013030290 PMC3968491 24262793

[36] Li LL Chu H Tao J Song D Tan M Wang SX. Lupus nephritis with obvious igA deposits in the kidneys. Am J Med Sci (2022) 363:174–84. doi: 10.1016/j.amjms.2020.11.032 34332968

[37] Anders HJ Kitching AR Leung N Romagnani P. Glomerulonephritis: immunopathogenesis and immunotherapy. Nat Rev Immunol (2023) 23:453–71. doi: 10.1038/s41577-022-00816-y PMC9838307 36635359

[38] Kaartinen K Safa A Kotha S Ratti G Meri S. Complement dysregulation in glomerulonephritis. Semin Immunol (2019) 45:101331. doi: 10.1016/j.smim.2019.101331 31711769

[39] Leon J Lestang MB Sberro-Soussan R Servais A Anglicheau D Fremeaux-Bacchi V. Complement-driven hemolytic uremic syndrome. Am J Hematol (2023) 98 Suppl 4:S44–56. doi: 10.1002/ajh.26854 36683290

[40] Bantis K Stangou M Kalpakidis S Hatziadamou M Daikidou DV Lioulios G. Systemic complement activation in anti-neutrophil cytoplasmic antibody-associated vasculitis and necrotizing glomerulonephritis. Nephrol (Carlton) (2021) 26:30–7. doi: 10.1111/nep.13747 32602136

[41] Nguyen ID Sinnathamby ES Mason J Urban B Neuchat EE Wenger DM. Avacopan, a novel competitive C5a receptor antagonist, for severe antineutrophil cytoplasmic autoantibody-associated vasculitis. Clin Drug Investig (2023) 43:595–603. doi: 10.1007/s40261-023-01298-z 37596445

[42] Sahu RK Xavier S Chauss D Wang L Chew C Taylor R. Folic acid-mediated fibrosis is driven by C5a receptor 1-mediated activation of kidney myeloid cells. Am J Physiol Renal Physiol (2022) 322:F597–610. doi: 10.1152/ajprenal.00404.2021 PMC9054266 35379003

[43] Tanaka S Portilla D Okusa MD. Role of perivascular cells in kidney homeostasis, inflammation, repair and fibrosis. Nat Rev Nephrol (2023) 19:721–32. doi: 10.1038/s41581-023-00752-7 37608184

[44] Van Der Veen SJ Van Kuilenburg ABP Hollak CEM Kaijen PHP Voorberg J Langeveld M. Antibodies against recombinant alpha-galactosidase A in Fabry disease: Subclass analysis and impact on response to treatment. Mol Genet Metab (2019) 126:162–8. doi: 10.1016/j.ymgme.2018.11.008 30473480

[45] Haddad G Lorenzen JM Ma H De Haan N Seeger H Zaghrini C. Altered glycosylation of IgG4 promotes lectin complement pathway activation in anti-PLA2R1-associated membranous nephropathy. J Clin Invest (2021) 131:e140453. doi: 10.1172/JCI140453 33351779 PMC7919733

[46] Karsten CM Pandey MK Figge J Kilchenstein R Taylor PR Rosas M. Anti-inflammatory activity of IgG1 mediated by Fc galactosylation and association of FcgammaRIIB and dectin-1. Nat Med (2012) 18:1401–6. doi: 10.1038/nm.2862 PMC3492054 22922409

[47] Nimmerjahn F Vidarsson G Cragg MS. Effect of posttranslational modifications and subclass on IgG activity: from immunity to immunotherapy. Nat Immunol (2023) 24:1244–55. doi: 10.1038/s41590-023-01544-8 37414906

[48] Hogan JJ Markowitz GS Radhakrishnan J. Drug-induced glomerular disease: immune-mediated injury. Clin J Am Soc Nephrol (2015) 10:1300–10. doi: 10.2215/CJN.01910215 PMC4491282 26092827

[49] West EE Kolev M Kemper C. Complement and the regulation of T cell responses. Annu Rev Immunol (2018) 36:309–38. doi: 10.1146/annurev-immunol-042617-053245 PMC7478175 29677470

[50] Del Pinto R Ferri C. The role of immunity in fabry disease and hypertension: A review of a novel common pathway. High Blood Press Cardiovasc Prev (2020) 27:539–46. doi: 10.1007/s40292-020-00414-w PMC7661400 33047250

[51] Sinuani I Beberashvili I Averbukh Z Sandbank J. Role of IL-10 in the progression of kidney disease. World J Transplant (2013) 3:91–8. doi: 10.5500/wjt.v3.i4.91 PMC3879528 24392313

[52] Sureshbabu A Muhsin SA Choi ME. TGF-beta signaling in the kidney: profibrotic and protective effects. Am J Physiol Renal Physiol (2016) 310:F596–606. doi: 10.1152/ajprenal.00365.2015 PMC4824143 26739888

[53] Su H Lei CT Zhang C. Interleukin-6 signaling pathway and its role in kidney disease: an update. Front Immunol (2017) 8:405. doi: 10.3389/fimmu.2017.00405 28484449 PMC5399081

[54] Rozenfeld PA De Los Angeles Bolla M Quieto P Pisani A Feriozzi S Neuman P. Pathogenesis of Fabry nephropathy: The pathways leading to fibrosis. Mol Genet Metab (2020) 129:132–41. doi: 10.1016/j.ymgme.2019.10.010 31718986

[55] Mehta T Buzkova P Kizer JR Djousse L Chonchol M Mukamal KJ. Higher plasma transforming growth factor (TGF)-beta is associated with kidney disease in older community dwelling adults. BMC Nephrol (2017) 18:98. doi: 10.1186/s12882-017-0509-6 28327102 PMC5359982

[56] Pandey MK Grabowski GA Kohl J. An unexpected player in Gaucher disease: The multiple roles of complement in disease development. Semin Immunol (2018) 37:30–42. doi: 10.1016/j.smim.2018.02.006 29478824

[57] Hawlisch H Kohl J. Complement and Toll-like receptors: key regulators of adaptive immune responses. Mol Immunol (2006) 43:13–21. doi: 10.1016/j.molimm.2005.06.028 16019071

[58] Reis ES Mastellos DC Hajishengallis G Lambris JD. New insights into the immune functions of complement. Nat Rev Immunol (2019) 19:503–16. doi: 10.1038/s41577-019-0168-x PMC6667284 31048789

[59] Zhang J Li Y Shan K Wang L Qiu W Lu Y. Sublytic C5b-9 induces IL-6 and TGF-beta1 production by glomerular mesangial cells in rat Thy-1 nephritis through p300-mediated C/EBPbeta acetylation. FASEB J (2014) 28:1511–25. doi: 10.1096/fj.13-242693 24344329

[60] Roth A Barcellini W D'sa S Miyakawa Y Broome CM Michel M. Sutimlimab in cold agglutinin disease. N Engl J Med (2021) 384:1323–34. doi: 10.1056/NEJMoa2027760 33826820

[61] Hillmen P Szer J Weitz I Roth A Hochsmann B Panse J. Pegcetacoplan versus eculizumab in paroxysmal nocturnal hemoglobinuria. N Engl J Med (2021) 384:1028–37. doi: 10.1056/NEJMoa2029073 33730455

[62] Hillmen P Hall C Marsh JC Elebute M Bombara MP Petro BE. Effect of eculizumab on hemolysis and transfusion requirements in patients with paroxysmal nocturnal hemoglobinuria. N Engl J Med (2004) 350:552–9. doi: 10.1056/NEJMoa031688 14762182

[63] Jayne DRW Merkel PA Schall TJ Bekker P Group AS. Avacopan for the treatment of ANCA-associated vasculitis. N Engl J Med (2021) 384:599–609. doi: 10.1056/NEJMoa2023386 33596356

